# Efficacy of integrated traditional Chinese medicine and Western medicine in the treatment of poststroke insomnia: A protocol for systematic review and meta-analysis of randomized controlled trials

**DOI:** 10.1097/MD.0000000000027396

**Published:** 2021-10-08

**Authors:** Wenwen Li, Cuncheng Liu, Ruiqi Wang, Ruxue Liu, Min Peng, Guomin Si

**Affiliations:** a Shandong University of Traditional Chinese Medicine, Jinan, Shandong, China; b Jiangxi University of Traditional Chinese Medicine, Nanchang, Jiangxi, China; c Shandong University, Jinan, Shandong, China.

**Keywords:** efficacy, integrated traditional Chinese medicine and Western medicine, poststroke insomnia, protocol, Western medicine

## Abstract

**Background:**

Western medicine has played an essential role in treating poststroke insomnia (PSI) in China, and traditional Chinese medicine therapy based on Chinese characteristics is also effective. Combined with China's national conditions, we plan to conduct this systematic review and meta-analysis to compare the efficacy of integrated traditional Chinese medicine and Western medicine (INTEGRATED TCM and WM) therapy and Western medicine alone for PSI.

**Methods:**

We will search the following 5 electronic databases: PubMed, Wanfang, Chinese biomedical literature database, the Chongqing VIP Chinese Science and Technology Periodical, and China national knowledge infrastructure. Randomized controlled trials that compared the efficacy of INTEGRATED TCM and WM with Western medicine alone in the treatment of PSI will be considered. Primary outcomes have Treatment effectiveness rate, and Pittsburgh sleep quality index. Secondary outcomes include traditional Chinese medicine syndrome score, Athens insomnia scale, the incidence of adverse reactions, and outcome follow-up. Based on the eligibility criteria, we will conduct literature screening and data extraction. The quality of the included literature will be evaluated using the Cochrane risk of bias tools. We will use Review Manager software (Version 5.3) for data synthesis and statistical analyses. If sources of heterogeneity exist, we will perform a subgroup analysis or sensitivity analysis. A funnel plot will be used to analyze publication bias.

**Results:**

This study will provide evidence-based medicine evidence for treatment of PSI with INTEGRATED TCM and WM in terms of its efficacy.

**Conclusion:**

This systematic review aims to provide new options for INTEGRATED TCM and WM treatment of PSI in terms of its efficacy.

## 1 Introduction

Poststroke insomnia (PSI) is a common type of stroke-related sleep disorders. It is a disease that first appears after stroke and reaches the diagnostic criteria of insomnia.^[1]^ Studies indicate that many factors, such as sleep and circadian rhythm disruptions,^[2]^ stroke lesion location,^[3]^ stroke severity,^[4]^ and age,^[5]^ may be related to the occurrence and development of PSI. It is an important public health problem in poststroke illness associated with a high prevalence rate, accounting for about 38.2%,^[6]^ which increases the risk of death and recurrence of stroke, and seriously affects the recovery and prognosis of patients,^[7–9]^ but it does not draw enough attention by both patients and doctors. Sleep occupies 1/4 to 1/3 of a lifetime in most humans and is an essential physiological process to maintain human health. Good sleep is the basis of high quality of life and the completion of various social activities. Improving patients’ and doctors’ awareness of the impact of sleep disorders on stroke and active treatment are beneficial to patients’ functional recovery and mental health, and reduce the recurrence rate and mortality rate of stroke.

China is a large population, with a heavy burden of stroke and a high incidence rate of stroke. The age of onset, the major lesion, and the type of the disease are different from those of other countries.^[10,11]^ It is of great significance to choose the appropriate treatment for stroke patients in China. At present, PSI pathogenesis remains incompletely understood. Western medicine mainly uses sedative-hypnotics, antianxiety, and antidepressants to improve the sleep status of patients with PSI,^[1]^ but it is easy to produce drug resistance and addiction. The clinical effect of traditional Chinese medicine (TCM) is positive, but the onset of efficacy occurs slowly. Integrated traditional Chinese medicine and Western medicine therapy can improve clinical effectiveness and reduce side effects. However, there is currently no meta-analysis on the effectiveness of Integrated traditional Chinese medicine and Western medicine in the therapy of patients with PSI. This study will use meta-analysis methods to evaluate the efficacy of the combined therapy in the treatment of PSI, aiming to provide evidence-based medicine for clinical practice.

## 2 Methods

### 2.1 Study registration

We will report the protocol based on the 2015 preferred reporting items for systematic reviews and meta-analysis protocols (PRISMA-P) guidelines.^[12]^

The protocol has been registered on the INPLASY website (https://inplasy.com/). The registration number is INPLASY202140028.

### 2.2 Ethics and dissemination

Our study does not directly contact with human or animal participants and is a systematic review and meta-analysis based on data from previously published randomized controlled trials (RCTs). Therefore, ethical approval is not required. We will disseminate the results in a peer-reviewed journal.

### 2.3 Eligibility criteria

Two review authors (Wenwen Li and Guomin Si) will independently assess every retrieved article. Eligibility criteria are as follows:

#### 2.3.1 Type of studies

All the studies are RCTs of PSI treated by a combination of Chinese and Western medicine. Studies involving animal experiments, case reports, basic research, reviews, non-RCTs will be excluded.

#### 2.3.2 Study subjects

The study participants are PSI patients with clear and standardized diagnostic criteria, regardless of case source, age, gender, and race. Patients with other types of sleep-related diseases such as excessive daytime sleepiness, restless leg syndrome, and sleep-disordered breathing will be excluded.

#### 2.3.3 Interventions

The control group only received conventional Western medicine treatment, and the experimental group was treated with TCM on the basis of the control group. The conventional Western medicine treatment may be different for each study, but whether or not they received TCM treatment was the only difference between the experimental group and the control group. TCM included single herb (including extract from single herb), Chinese patent medicine or Chinese medicine compound prescription, regardless of drug preparation (e.g., decoction, oral liquid, tablet, capsule, pill, powder, granule, injection, or plaster), route of administration (e.g., oral, topical, intramuscular or intravenous injection), dosage, and regimen of herbs.

#### 2.3.4 Outcomes

Primary outcomes: Treatment effective rate, Pittsburgh sleep quality index.

Secondary outcomes: TCM syndrome score, Athens insomnia scale, the incidence of adverse reactions, outcome follow-up.

### 2.4 Literature search

Electronic databases will be systematically searched for relevant reports from their inception to August 24, 2021: PubMed, Wanfang, Chinese biomedical literature database (CBM), the Chongqing VIP Chinese Science and Technology Periodical (VIP), and China national knowledge infrastructure (CNKI). We will use the following combined free text terms and Medical Subject Headings (Mesh) terms as follows: “PSI” or “Insomnia after stroke”, “Sleep Initiation and Maintenance Disorders” or “Disorders of Initiating and Maintaining Sleep”, “stroke” or “Strokes”. Using PubMed as an example, the initial search strategy is shown in Table 1. We will also identify additional relevant articles by manually reviewing the references of the included papers.

**Table 1:** Search strategy of the Pubmed.

Number	Search terms
#1	“Sleep Initiation and Maintenance Disorders”[Mesh]
#2	Disorders of Initiating and Maintaining Sleep [Title/Abstract] OR DIMS[Title/Abstract] OR Early Awakening[Title/Abstract] OR Awakening, Early[Title/Abstract] OR Insomnia[Title/Abstract] OR Sleep Initiation Dysfunction[Title/Abstract] OR Dysfunction, Sleep Initiation[Title/Abstract] OR Sleeplessness[Title/Abstract]
#3	#1 OR #2
#4	“Stroke”[Mesh]
#5	Strokes[Title/Abstract] OR Cerebrovascular Accident[Title/Abstract] OR CVA[Title/Abstract] OR Apoplexy[Title/Abstract] OR Vascular Accident, Brain[Title/Abstract] OR Brain Vascular Accident[Title/Abstract]
#6	Poststroke insomnia[Title/Abstract] OR Insomnia after stroke[Title/Abstract]
#7	#4 OR #5 OR #6
#8	Randomized controlled trial[Publication Type] OR randomized[Title/Abstract] OR placebo[Title/Abstract]
#9	#3 AND #7 AND #8

### 2.5 Data collection and analysis

#### 2.5.1 Selection of studies

**Figure 1:**
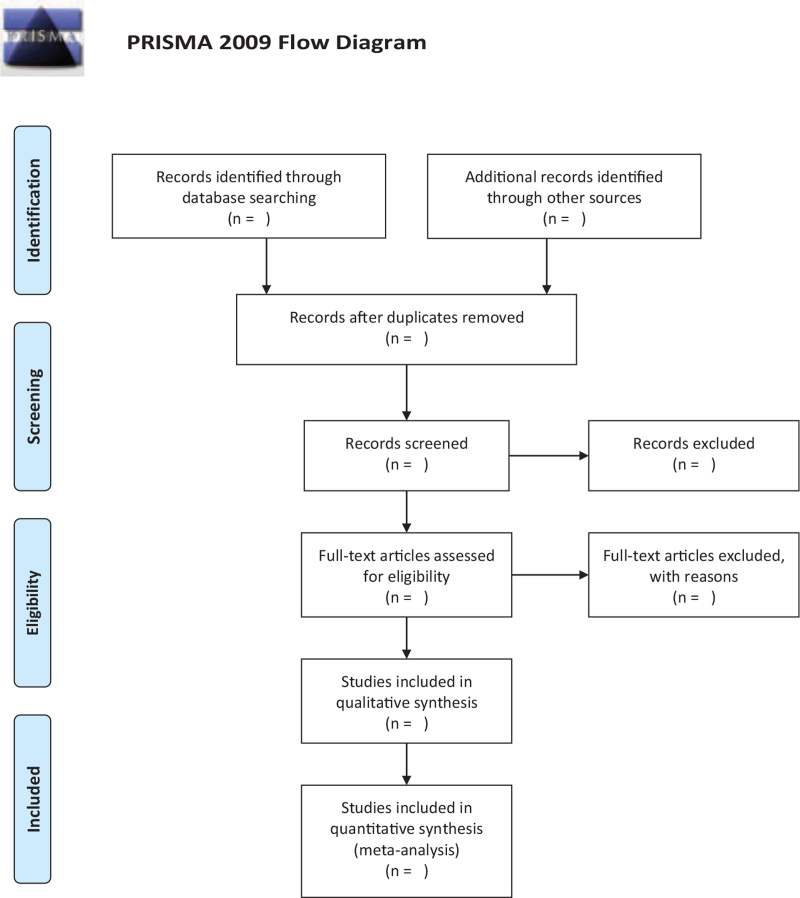
The flowchart of the screening process.

The databases are searched according to the search strategy, and all the retrieved studies are imported into Endnote X9 software (Thomson Research Soft, Stanford, Connecticut) for screening. After eliminating duplicates, 2 independent researchers (Wenwen Li and Guomin Si) further read the titles and abstracts to exclude noncompliant literature. The literature included after the initial screening is carefully read in full and then screened again to decide on the final inclusion. Any objections during the screening process are referred to a third party (Min Peng) for decision. The screening process is shown in Figure 1.

#### 2.5.2 Data extraction and management

We will perform data collection and management according to Cochrane Review procedures. The extracted information will include: the first author, year of publication, funding source, sex, age, duration of disease, sample size (including experimental group and control group), interventions, course of treatment, and outcome indicators. Based on the characteristics of the information extracted, an excel sheet is prepared for data collection. The same 2 review authors (Wenwen Li and Guomin Si) will independently extract data. In case of disagreement, they will be discussed or decided by an independent third review author (Min Peng). If data in the literature is incomplete, we will try to contact the authors of the original study to complete the information. If the author cannot be contacted, we will exclude the study due to a lack of essential data.

#### 2.5.3 Risk of bias assessment

Two researchers (Cuncheng Liu and Ruiqi Wang) will independently assess the methodological quality of each paper by using the Cochrane risk of bias tools. The main items include random sequence generation, allocation concealment, blinding, incomplete outcome data, selective reporting, and other bias. Each item is divided into “low risk”, “unclear risk”, and “high risk”. Where differences exist, we will reach a consensus through discussions with third parties (Ruxue Liu). The risk of bias assessment map will be created through Revman software (The Nordic Cochrane Center, The Cochrane Collaboration, 2014, Copenhagen, Denmark).

#### 2.5.4 Data analysis

Review Manager software version 5.3 (The Nordic Cochrane Center, The Cochrane Collaboration, 2014, Copenhagen, Denmark) will be used for meta-analysis. Relative risk (RR) with 95% confidence interval (CI) will be calculated for dichotomous data, and mean difference (MD) with 95% CI will be calculated for continuous data. *P* < .05 is a statistically significant difference for all analyses. The level of statistically significant heterogeneity will be set at *P* < .1. In addition, the *I*^*2*^ value will be used to analyze the heterogeneity quantitatively. If *P* > .1 and *I*^*2*^ ≤ 50%, meta-analysis will adopt fixed-effect; If *P* ≤ .1 or *I*^*2*^ > 50%, meta-analysis will adopt random-effect. If the random-effect is used, we will perform subgroup analysis or sensitivity to clarify the source of heterogeneity. If quantitative synthesis is not possible, we will perform a descriptive analysis.

#### 2.5.5 Subgroup analysis

We will explore the sources of heterogeneity through subgroup analysis based on age, gender, duration of disease, different interventions, duration of treatment, etc.

#### 2.5.6 Sensitivity analysis

To determine whether the decisions at each step are robust and impact the combined results, a sensitivity analysis will be conducted. Each time a study is excluded, a new meta-analysis is conducted separately to see if the effect size changes. We will also use this method to explore the sources of heterogeneity if there is significant heterogeneity in the studies.

#### 2.5.7 Publication bias

If more than 10 studies are eventually included, we will use a funnel plot to analyze publication bias and interpret the results with caution.

#### 2.5.8 Grading the quality of evidence

In this study, we will use the internationally accepted Grades of Recommendations Assessment, Development and Evaluation (GRADE) system to assess the quality of the evidence. The quality of evidence is classified into 4 levels: high quality, medium quality, low quality, and very low quality.

## 3 Discussions

The incidence of stroke increases with age.^[13]^ As China enters an aging society, there will be more people suffering from cerebrovascular disease. PSI usually occurs during the acute phase of stroke^[3]^ and is a common type of sleep disorders after stroke. A systematic review and meta-analysis^[6]^ shows that the probability of insomnia after a stroke has reached about one-third, and PSI often causes more severe consequences. Therefore, effective treatment methods are extremely essential to improve the survival rate and quality of life of patients. One study has shown that the use of Gamma-aminobutyric acid (GABA) receptor agonists in the acute phase of a stroke may be harmful,^[14]^ and other studies have shown that the use of benzodiazepines can increase the risk of cognitive impairment and stroke.^[15,16]^ To compensate for the lack of Western medicine alone and reduce the possible risks, we believe that a combination of Western and Chinese medicine can be used. With more and more research linking Chinese medicinal herbs and their extracts to various modern diseases,^[17]^ TCM is gaining more and more recognition in the international medical field. TCM believes that the cause of insomnia is mainly related to the imbalance of yin-yang. As PSI is secondary to the stroke, the disease also reflects the characteristics of the recovery and the sequelae of the stroke period, that is, a mixture of xu and shi. Therefore, the treatment should be directed at yin-yang and xu-shi. Good results have been achieved in terms of both specific medication and prescription. Herbs often used include Yuan Zhi, *Ziziphus jujuba* Mill, Albiziae Cortex, Fu Shen, Shou Wu Vine, and platycladi seed. In terms of prescription, the commonly used Chinese medicine prescriptions include Sour Jujube decotion, Tian-wang-bu-xin-dan, Huanglian Ejiao decoction, and Jiaotai Pill. Modern pharmacological studies confirmed the sedative and hypnotic effects of Chinese medicine and its active ingredients. They expounded the action ways of Chinese medicine in regulating central neurotransmitter affecting sleep-related cytokines, and improving the structure of the central nervous system.^[18,19]^

However, due to the complex mechanism of TCM therapy, its main application area are still in China, which limits the development of TCM. It is this uniqueness of TCM that makes our research meaningful.

## Author contributions

Wenwen Li, Cuncheng Liu, and Guomin Si proposed this research and wrote the first draft of the manuscript. Guomin Si and Peng Min provided research funds and supervised the whole process. Wenwen Li, Cuncheng Liu, Ruxue Liu, and Ruiqi Wang collected resources and conducted data analysis. All authors participated in the finalization of the manuscript and approved the manuscript.

**Conceptualization**: Wenwen Li, Cuncheng Liu, Guomin Si.

**Data curation**: Wenwen Li, Min Peng, Guomin Si.

**Formal analysis**: Ruiqi Wang, Guomin Si.

**Funding acquisition**: Min Peng, Guomin Si.

**Investigation**: Wenwen Li, Cuncheng Liu, Ruxue Liu.

**Methodology**: Ruiqi Wang, Ruxue Liu.

**Project administration**: Wenwen Li, Cuncheng Liu, Guomin Si.

**Resources**: Wenwen Li, Cuncheng Liu.

**Software**: Wenwen Li, Cuncheng Liu, Ruiqi Wang.

**Supervision**: Min Peng, Guomin Si.

**Validation**: Ruiqi Wang, Ruxue Liu.

**Visualization**: Wenwen Li, Cuncheng Liu.

**Writing – original draft:** Wenwen Li, Guomin Si.

**Writing – review & editing:** Cuncheng Liu, Guomin Si.
